# Impairment of Gradual Muscle Adjustment during Wrist Circumduction in Parkinson's Disease

**DOI:** 10.1371/journal.pone.0024572

**Published:** 2011-09-02

**Authors:** Carolien M. Toxopeus, Bauke M. de Jong, Gopal Valsan, Bernard A. Conway, Johannes H. van der Hoeven, Klaus L. Leenders, Natasha M. Maurits

**Affiliations:** 1 Department of Neurology, University Medical Center Groningen, University of Groningen, Groningen, The Netherlands; 2 Bioengineering Unit, University of Strathclyde, Glasgow, United Kingdom; Philadelphia VA Medical Center, United States of America

## Abstract

Purposeful movements are attained by gradually adjusted activity of opposite muscles, or synergists. This requires a motor system that adequately modulates initiation and inhibition of movement and selectively activates the appropriate muscles. In patients with Parkinson's disease (PD) initiation and inhibition of movements are impaired which may manifest itself in e.g. difficulty to start and stop walking. At single-joint level, impaired movement initiation is further accompanied by insufficient inhibition of antagonist muscle activity. As the motor symptoms in PD primarily result from cerebral dysfunction, quantitative investigation of gradually adjusted muscle activity during execution of purposeful movement is a first step to gain more insight in the link between impaired modulation of initiation and inhibition at the levels of (i) cerebrally coded task performance and (ii) final execution by the musculoskeletal system. To that end, the present study investigated changes in gradual adjustment of muscle synergists using a manipulandum that enabled standardized smooth movement by continuous wrist circumduction. Differences between PD patients (N = 15, off-medication) and healthy subjects (N = 16) concerning the relation between muscle activity and movement performance in these groups were assessed using kinematic and electromyographic (EMG) recordings. The variability in the extent to which a particular muscle was active during wrist circumduction – defined as muscle activity differentiation - was quantified by EMG. We demonstrated that more differentiated muscle activity indeed correlated positively with improved movement performance, i.e. higher movement speed and increased smoothness of movement. Additionally, patients employed a less differentiated muscle activity pattern than healthy subjects. These specific changes during wrist circumduction imply that patients have a decreased ability to gradually adjust muscles causing a decline in movement performance. We propose that less differentiated muscle use in PD patients reflects impaired control of modulated initiation and inhibition due to decreased ability to selectively and jointly activate muscles.

## Introduction

Successful execution of purposeful movement requires a motor system in which selective initiation and inhibition of successive movements are adequately modulated [1], [2]. This requires controlled and gradual adjustment of (synergistic) muscles [2], [3]. These co-activations, or muscle synergies, defined as co-variations of elemental variables [4], enable the performance of a large variety of movements in different directions while only a limited number of muscles is available. The basal ganglia (BG) play a key role in modulating initiation and inhibition of movement since they inhibit undesired motor activity and select appropriate muscle synergies [5], [6]. Such a specific role of the BG and interconnected circuitry in continuous modulation of initiation and inhibition is further inferred from extrapyramidal movement disorders such as Parkinson's disease (PD). BG dysfunction in PD causes impaired initiation and inhibition of movement leading to movement problems such as hesitation, propulsion and rigidity [7]–[9]. Yet, it is not clear how pathophysiological changes in the BG exactly lead to the impaired ability to perform purposeful movement. Execution of the latter is based on smooth modulation of muscle activity. The main aim of the present study was therefore to quantitatively investigate how changes in the control of muscle synergies, on the neurophysiologic level, are related to a decline in smooth movement performance in PD patients.

PD is a degenerative movement disorder of the central nervous system (CNS) caused by degeneration of pigmented brain stem nuclei, including the dopaminergic substantia nigra pars compacta [10] which eventually results in changes in the activity of neural pathways within the basal ganglia-thalamocortical circuits controlling movement [7], [9]–[12]. Clinically, PD is characterized by muscle rigidity, tremor, bradykinesia and abnormalities in posture and balance [9], [10]. At task level, impaired initiation of movement in PD is illustrated by hesitation in the onset of walking as well as “freezing of gait”; a clinical expression of initiation problems in which patients are suddenly unable to move and seem to be “stuck” to the floor [13]. On the other hand, at single-joint level, impaired movement initiation [14] is also associated with insufficient inhibition of the antagonist muscle [15]. Additionally, besides deficits in explicitly starting and stopping of movement, PD patients may also exhibit impairments of purposeful movement execution. For example, they show more undershoot in reaching the target and have impairments in the coordinated movement of joints compared to healthy subjects [16], [17]. Hypothetically, PD-related changes in BG functionality cause impaired selection of appropriate muscle synergies which may become particularly evident during tasks that demand highly tuned changes in timing and magnitude of muscle activity.

Investigation of gradually tuned muscle activity is optimally done by using a movement task that particularly requires synergistic muscle activity rather than one focusing on a single element of movement such as wrist flexion or extension [14], [18]. The present study, therefore, employed a task consisting of continuous wrist circumduction. In such a task, indeed, muscles are not pure agonists or antagonists but instead work together as synergists in a gradually adjusted manner. By using a continuous wrist circumduction task we aimed to investigate 1) differences in continuous movement requiring gradual muscle adjustment between Parkinson patients and healthy subjects and 2) the relation between muscle activity and movement performance in these groups using both kinematic and electromyographic (EMG) recordings. Although tasks consisting of continuous circular movements have been used in previous studies on motor control in PD, these studies often used circular tracking, or drawing tasks involving the arm and aimed to investigate impaired multi-joint coordination [19], [20] or were focused on changes in movement timing in PD [21]. The multi-joint task-design in these studies, however, is not particularly focused on gradual adjustment of opposite muscles.

In the present study we used a circular *single-joint* movement task in combination with a manipulandum and visual feedback, which enabled standardized execution of a continuous movement. Therefore, our modification of the circular movement task allows to specifically investigate changes in gradual adjustment of opposing muscles in PD patients. We hypothesized that PD patients have impaired gradual muscle adjustment during continuous wrist circumduction causing a decline in smooth movement performance.

## Materials and Methods

### Ethics Statement

The study was approved by the Medical Ethical Committee of the University Medical Center Groningen. Subjects gave written informed consent in accordance with the Declaration of Helsinki (2008) prior to participation.

### Subjects

Sixteen healthy elderly aged 60–70 years and seventeen patients with idiopathic PD experiencing mild to moderate clinical symptoms participated in the present study. Patients were assessed by the Unified Parkinson's Disease Rating Scale (UPDRS) [22] and Hoehn Yahr disability scale [23] (off-medication). Patients had to have a stable response to medications and were requested not to take their morning dose of levodopa, or dopamine agonists (overnight withdrawal) in order to reduce medication effects. Subjects had to be right handed (assessed by the Annett Handedness Scale [24]). Exclusion criteria for both groups were a history of epileptic seizures, head injury, neurological diseases (for patients: other than PD), psychiatric diseases or the use of any type of medication affecting the CNS. Subjects who had a Mini Mental State Examination (MMSE [25]) score below 25 were excluded. Additionally, PD patients who suffered from the tremor dominant type of PD were excluded since (i) we aimed to obtain a maximally homogeneous group of PD patients and tremor-dominant PD might be regarded as a PD subtype [26], [27] and (ii) when investigating muscle activity inclusion of patients with muscle activity patterns dominated by tremor would interfere with the results since we were not specifically interested in measuring tremor effects. Patients with Parkinsonism other than PD were also excluded from participation in the study to obtain a maximally homogeneous patient group.

Of the seventeen PD patients, one patient was excluded from all analyses due to use of anti-Parkinsonian medication during the experiment. Another patient was not able to execute the circle task (mean RT up to 2 seconds) and was therefore also excluded from further analyses. Due to a technical failure, EMG data was not recorded for one patient, which was the reason to analyze only the obtained kinematic data. For two subjects (one patient and one healthy subject) only three out of the four blocks of circle movements were recorded due to a technical problem (these data were included in all data analyses). Thus, fifteen patients were included in the kinematic analysis and fourteen patients in the EMG analysis. Data from sixteen healthy subjects (age range: 60–70, mean: 64.5, standard deviation (SD): 2.8, male (6)) and fifteen patients (age range: 38–69, mean: 59.1, SD: 7.9, male (10); all with idiopathic PD) eventually entered the analysis (for patient characteristics see table 1). The youngest patient was clinically similar to the older patients and was included, as she did not have an exceptional trait such as e.g. a genetic mutation. Overall, differences in the dominant side of Parkinsonian symptoms between patients were subtle. According to the combined UPDRS scores for rigidity and bradykinesia for the upper extremity, which represent important measures regarding motor control and muscle co-activation in patients, eight patients had a higher score for the right side, while three patients had a higher score for the left side and four had symmetrical scores. Healthy subjects were significantly older than patients (T-test, p = 0.028). Practically, this was the result of the healthy elderly being included for another study and for ethical reasons, we did not want to include more subjects than required.

**Table 1:** Clinical details of patients with Parkinson's disease.

Age (years ± SD)	59.1±7.9
Sex	10 male, 5 female
Disease duration (years ± SD)	4.0* (8.0)
UPDRS motor score	23.0* (11.0)
UPDRS rigidity (upper extremity)	3.0* (4.0)
UPDRS bradykinesia (upper extremity)	8.0* (5.0)
Rigidity and Bradykinesia	R>L = 8 (mean difference = 3);
(UPDRS; upper extremity)	equal = 4; L>R = 3 (mean difference = 2)
Hoehn and Yahr stage	2.0* (1.0)

Clinical details of patients with Parkinson's disease. *  =  median (interquartile range) due to non-normality. R =  right, L =  left. All scores are off medication.

### Experimental set-up

**Figure 1:**
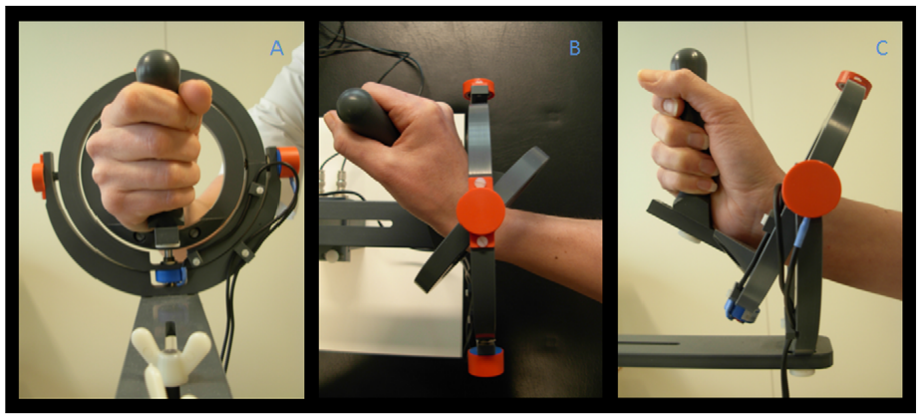
Photograph of the wrist manipulandum. The construction consists of two concentric rings that move around perpendicular axes and allow two degrees of freedom for wrist movement: wrist flexion-extension, ulnar-radial deviation and all combinations thereof. Subjects were seated with the right arm supported by an armrest; a: (frontal view) neutral position (origin), the right hand was positioned in a vertical plane, holding the grip of the manipulandum; b: (top view) full wrist extension and c: (side view) full radial deviation.

All subjects performed a visually guided circular tracking task using a manipulandum similar to the manipulandum used by Hoffman and Strick for centre out step-tracking tasks [28] (fig. 1). Subjects were comfortably seated with the right arm supported by an armrest. The right hand was initially positioned in the vertical plane, holding the grip of the manipulandum with the thumb on top. In order to minimize differences in force used to grip the manipulandum between subjects, we explicitly instructed subjects to loosely hold the manipulandum and not to squeeze the grip. This instruction was given prior to the experiment and was repeated during the breaks between task blocks. The wrist joint was positioned in the center of the two concentric rings forming the fundamental components of the device. The manipulandum is a joystick-like device that permits movement in two perpendicular planes allowing wrist flexion-extension, wrist ulnar-radial deviation and all combinations thereof. In the employed task, continuous wrist circumduction is used to create circular trajectories of the hand about the wrist. This set-up facilitated subjects to make continuous circles using the distal radio-ulnar (wrist) joint exclusively. However, due to asymmetry of the wrist joint, maintaining the same grip position implies that wrist circumduction produces an ellipsoidally shaped movement trajectory instead of a symmetrical circle. To ensure that all subjects would be focused on smooth execution of continuous wrist circumduction rather than on making a perfect circle-shaped movement trajectory, which would cause corrective movements interfering with smoothness of movement, the angular position of the hand in the device was projected onto a virtual circle before being displayed as a cursor on the screen. This implies that subjects did not receive feedback on the actual shape of the movement trajectory but, instead, as long as they moved around the neutral position, subjects saw their movement as circle shaped on the same trajectory as the moving target. Additionally, subjects were trained to make maximally wide circumduction movements around the neutral position. To determine the angular position two potentiometers (θ and ϕ) are integrated in the manipulandum. The output from the potentiometers enables the derivation of kinematic parameters for comparison of task execution between groups. The task was presented to the subjects on a screen placed in front of them (distance ±50 cm). A warning cross was displayed in the center of the screen, with the target (2×2 cm open square) at a starting position located at 3 ‘o clock (maximum wrist extension). A cursor representing wrist angular position (5×5 mm closed square) could be moved by moving the hand about the wrist joint while holding the manipulandum. Subjects were instructed to move the cursor into the target zone and wait for the warning cross to disappear. One second after its disappearance, the target started to move at constant speed (1 circle/1.8 s = 3.5 rad/s), either clockwise (CW) or counter clockwise (CCW). Subjects were instructed to follow this moving target as smoothly and accurately as possible. The circle task was presented in 4 blocks with rest periods of 20 seconds in between. Each block consisted of 4 alternating runs of CW and CCW circles in the same order. One run of circle movements consisted of 10 continuous circular movements. The task was practiced for at least one full block to ensure every subject understood and was able to execute the task during the recording session. The kinematic data was collected using an analog-to-digital converter board (*Power 1401, Cambridge Electronic Design (CED), Cambridge, U.K.*). Data was digitized on-line at a sampling rate of 100 Hz using Spike 2 (*CED, UK*).

#### EMG recording

EMG electrodes were placed on the right arm to provide a measure of muscle activity during continuous wrist circumduction. Five bipolar electrode pairs were placed on the lower arm muscles (m. Extensor carpi radialis longus (m. ECRL), m. Extensor carpi radialis brevis (m. ECRB), m. Flexor carpi radialis (m. FCR), m. Extensor carpi ulnaris (m. ECU) and m. Flexor carpi ulnaris (m. FCU)). A reference electrode was placed on the dorsal side of the left hand. In order to improve skin conductance, the skin was pre-treated with a scrub gel. Electrodes were attached approximately 1.5 cm apart and placed longitudinally with respect to the muscle fibres. The different muscles were identified by palpation and by using the EMG of maximum voluntary contractions (MVC) towards the specific pulling direction of each individual muscle. These MVCs were not stored for further use. Although muscle-crosstalk can not be excluded when using surface-EMG, we tried to reduce cross-talk by verifying that movement towards the pulling direction elicited activity in the EMG channel belonging to that muscle, specifically. EMG data of healthy subjects was recorded at 2000 Hz using Onyx software (Silicon Biomedical Instruments BV, Westervoort, The Netherlands). EMG data of patients was recorded at 5000 Hz using Brain Vision Recorder software (Brain Products GmbH Munchen, Germany). EMG sampling rates differed between groups since data for PD patients were recorded at another location. Preparation of the experimental set-up including the attachment of EMG electrodes was performed by the same investigator (CMT).

### Data analysis

#### Kinematic parameters

The kinematic data was exported from Spike2 to Matlab (Mathworks, Natrick, MA, USA) and further analyzed individually for each subject. Three parameters were derived from the kinematic data: ‘radial position’ (RP), ‘speed’ and ‘angular distance’ (AD) and calculated per individual for each direction (CW and CCW). All data was averaged over bins of 1 degree with an entire circle consisting of 360 degrees. For all three parameters, the standard deviation (SD) over all 80 executed circles was calculated for each bin (with regard to cursor position) and for each direction. Reaction time (RT) was determined as a measure of initial movement delay by calculating the mean time between the start of the moving target and the start of the movement of the subject cursor, over all cycles of 10 consecutive circles.

Radial position (RP) was defined as the actual radial position of the hand within the manipulandum (relative to the neutral position of the manipulandum) and was used to determine the shape of the actual wrist trajectory. RP was calculated from the angular displacements θ and ϕ in radians as follows: 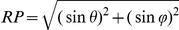



The actual radius of the movement depends on the individual range of motion of an individual (determined by hand size and joint excursion) and was not incorporated in the calculation of RP. Thus, RP yields the actual shape of the wrist trajectory, but at a normalized size. The mean RP and its SD (RP variability) for all 80 executed circles were calculated for each bin and for each direction. For each individual subject it was verified whether the wrist trajectory encircled the neutral position at all times, before including this subject into the analysis. Speed (rad/s) was calculated as the numerical first-order derivative of angular displacement in the circular movement i.e., as the difference between two subsequently sampled angular positions divided by the sample length (0.01 s). Similar as for RP, the mean subject cursor speed and SD (speed variability) were calculated for each bin (with regard to cursor position, not to target position) and for each direction. AD was calculated as the absolute difference between target and subject cursor positions (in degrees). This parameter represents how well the subject was able to stay on target. Notice that this parameter can take on values larger than 360 when subjects fall behind more than one circle. The mean AD and its SD (AD variability) over all 80 executed circles were calculated for each bin and for each direction. Polar plots were made for the three parameters RP, speed and AD per individual.

Due to anatomical limitations in wrist joint mobility and the requirement for subjects to grip the manipulandum, the shape of the actual movement trajectory deviates from a perfect circle. To quantify this deviation, the correlation between the two Cartesian coordinates of the actual trajectory (employing mean RP) was calculated for each subject and each direction. A perfectly circular trajectory would correspond to a correlation of zero since the points on a circle have no linear relationship. Positively skewed ellipsoidal trajectories (long axis tilted to the right) will yield positive correlations and negatively skewed ellipsoidal trajectories (long axis tilted to the left) will yield negative correlations. A similar analysis was performed for mean (absolute) speed and for mean AD. Resulting parameters are referred to as RP profile, speed profile and AD profile. The latter analyses allow the quantification of consistency of circumduction movement execution over the four quadrants in one number. In addition, polar plots of the three parameters were made to facilitate visual comparison between the two groups of subjects.

#### EMG

**Figure 2:**
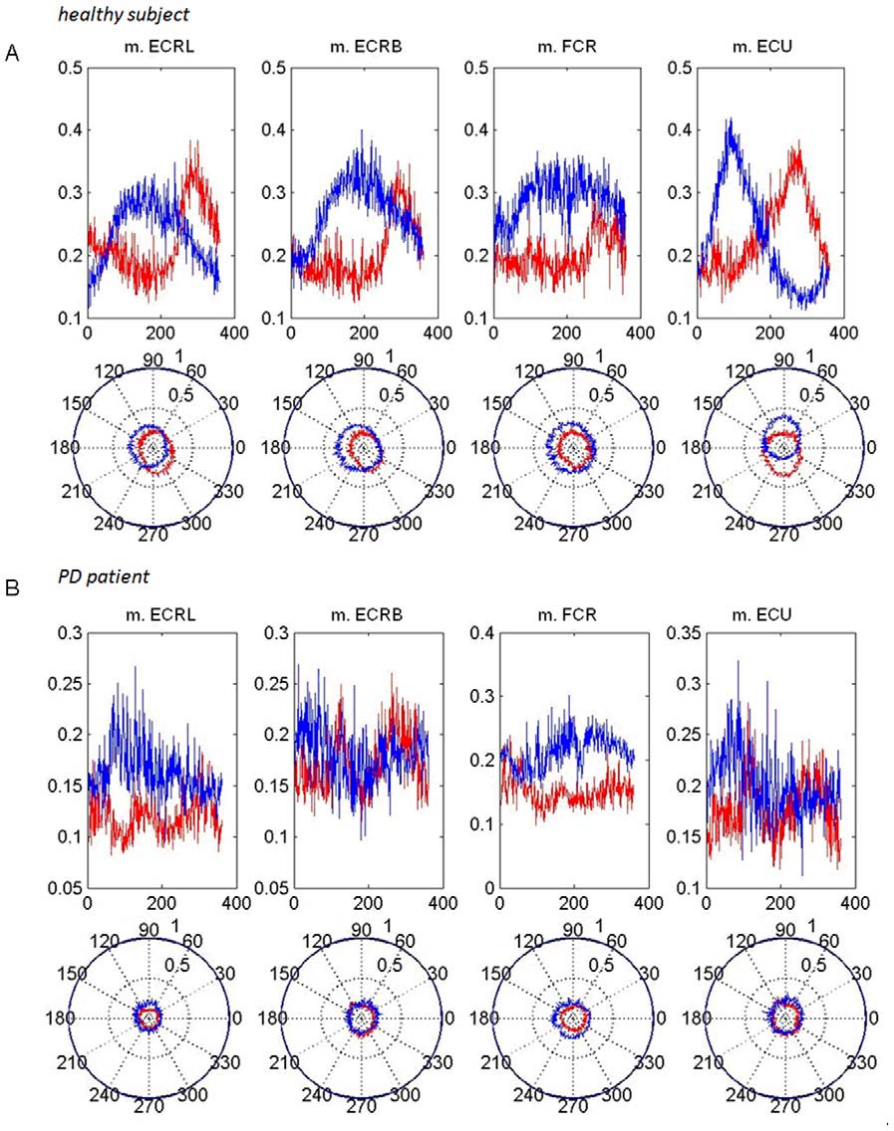
Examples of individual EMG data. EMG (mean) data of one healthy subject (A) and one PD patient (B) showing electromyographic (EMG) data of four lower arm muscles (m. extensor carpi radialis longus, (m. ECRL), m. extensor carpi radialis brevis (m. ECRB), m. flexor carpi radialis (m. FCR) and m. extensor carpi ulnaris (m. ECU) as a function of angle along the circumduction trajectory. Top: linear plots of mean smoothed, rectified and scaled EMG (note differences in scale for both subjects). x-axis = position on the circle (in degrees), y-axis  =  EMG. Notice that the scaled EMG has been averaged over all cycles, so that maximum values smaller than 1 are attained. Bottom: polar plots of mean smoothed, rectified and scaled EMG; the latter data were used to quantify differentiated muscle use by fitting ellipses. blue plots: CW, red plots: CCW.

**Figure 3:**
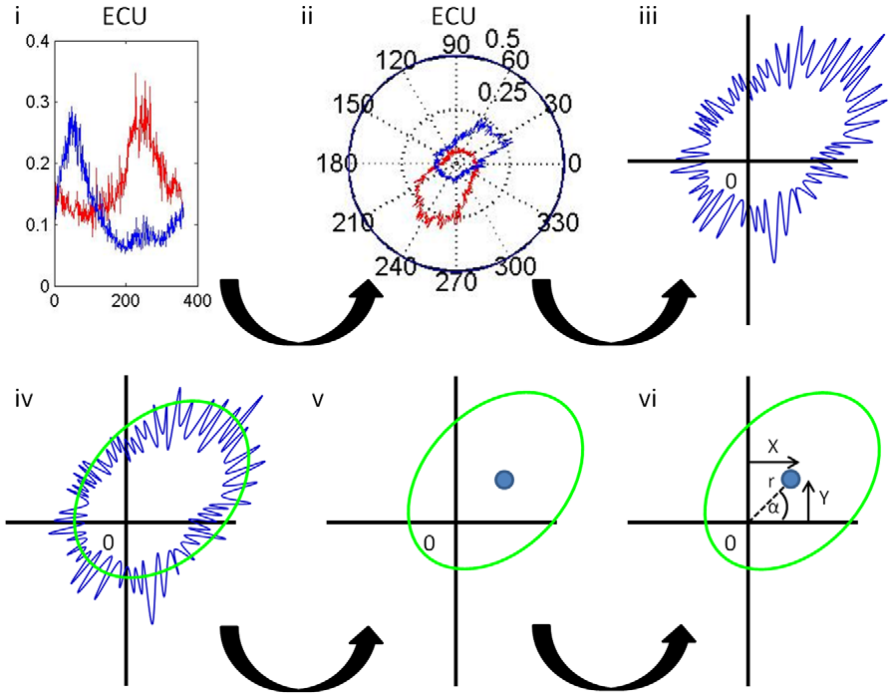
Quantification of muscle activity patterns. i–iii: Example of individually filtered, rectified and scaled EMG data (m. extensor carpi ulnaris (m. ECU), blue =  clockwise (CW), red =  counter clockwise (CCW); the scaled EMG data is indicated along the 75 degree axis. iv–v: Scaled EMG power was fitted to an ellipse (resulting in ‘EMG ellipse’); vi: By transforming the coordinates (X,Y) of the center of the EMG ellipse to polar coordinates, the parameters differentiated muscle activity (DiffAct) and angle at maximum activity were obtained (Angle_max_). DiffAct and Angle_max_ were used to compare differentiated muscle use between groups, muscles and direction.

The EMG data was exported to Matlab. EMG data was high-pass filtered using a Butterworth Zero Phase shift filter with a cut-off of 10 Hz and rectified using a custom made script. The EMG data was then down sampled to the frequency of the kinematic data (100 Hz). Next, the EMG as a function of time was transformed to EMG as a function of angle. The filtered and rectified EMG was then scaled by dividing it by its maximum over the 360 degrees of the circle. This provided a scaled EMG (with a value between 0 and 1) for each position on the circle. Subsequently, the mean scaled EMG along the trajectory (over all blocks) was calculated for each direction and muscle separately. Finally, plotting the mean scaled EMG in a polar plot enabled visualization of the variability in the extent to which a particular muscle was active during wrist circumduction for each individual subject and for each muscle. These plots enabled visual assessment of activity modulation of a specific muscle along the movement trajectory. Visual comparison showed that healthy subjects had a more ellipsoidal and eccentric EMG shape in the polar plot indicating that individual muscles were more active during a specific movement phase, which can be defined as increased ‘muscle activity differentiation’ (see examples in fig. 2A–B). In order to quantitatively compare muscle differentiation along the trajectory between muscles and groups, we applied data reduction, by employing the ellipsoidal shape (fig. 3.i–ii) of the plotted scaled EMG. A second script (Matlab) was used to fit an ellipse to the scaled EMG [*29*] (fig. 3.iii–v). The mathematical description of the fitted ellipse (center and axes) provides condensed information on the changes in the scaled EMG of a specific muscle along the circumduction movement trajectory. To enable statistical analysis of the EMG data two parameters were derived from the fitted ellipse; 1) the distance of the center of the ellipse to the origin (0,0) as a measure of the extent of differentiated muscle activity (DiffAct) (the eccentricity of the ellipse) and 2) the part of the trajectory in which the activity of the muscle is maximal (Anglemax). These parameters were obtained by transforming the Cartesian coordinates X and Y of the center of the ellipse to polar coordinates (see also fig. 3vi):
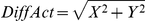


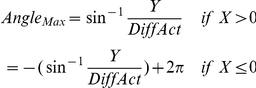



If Angle_max_ was negative another 2π was added to further facilitate statistical comparison. Together, DiffAct and Angle_max_ were used to quantify differentiation of muscle activity patterns, thereby enabling the quantification of gradual adjustment of muscle activity during continuous circle movement.

#### Statistics

The statistical analysis was performed using SPSS 16 (SPSS, Inc., Chicago IL). Separate repeated measures ANOVAs were used to test for general significant differences for all kinematic parameters including RT and all EMG parameters, separately. RT was compared between groups since a delay in movement onset could underlie higher values for AD or movement variability. The between-subject factor was group (2 levels: patients and healthy subjects) and the within-subject factor was direction (2 levels: CW and CCW). For the EMG analysis, muscle was added as a second within-subject factor (5 levels: m. ECRL, m. ECRB, m. FCR, m. ECU and m. FCU). Here, the dependent variables were DiffAct and Anglemax as derived from the fitted EMG ellipse. Main muscle effects for these EMG parameters were post-hoc tested by either parametric or non-parametric pair-wise tests. Tests for normality were performed using the Shapiro-Wilk test and Q-Q plots.

Additionally, to investigate the relations between the quality of movement execution as expressed in the kinematic parameters and the extent of differentiated muscle use as derived from the EMG we used correlations. Here, Pearson's correlation coefficient was used for normally distributed data and Spearman's correlation coefficient was used when data was not normally distributed. The significance level for all tests was set at α = 0.05.

## Results

### Kinematic parameters

#### We first generally describe the kinematic results

**Figure 4:**
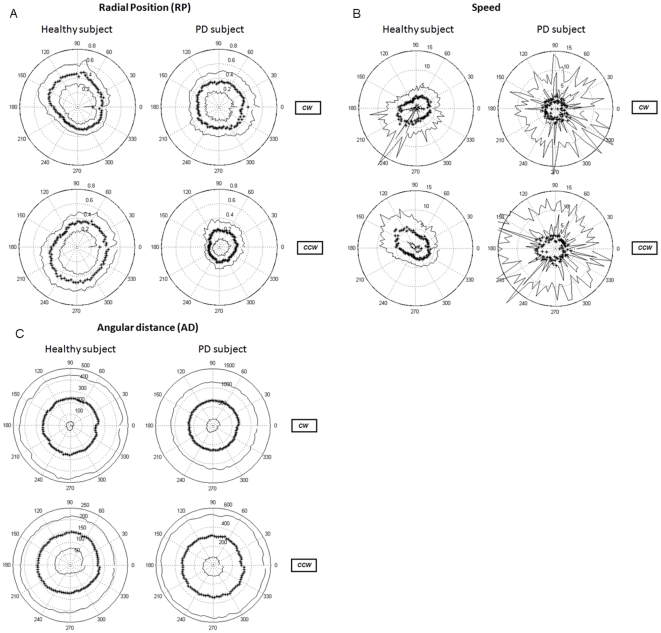
Examples of individual (mean) data in polar plots. Left: healthy subject, right: PD patient. Top: clockwise (CW), bottom: counter clockwise (CCW). Star line: mean, solid lines: outer line: mean + SD, inner line: mean - SD. Angles are indicated in degrees along the outer circle. The scale for each individual plot is indicated along the 75 degree axis. A: radial position (RP, dimensionless), B: speed (rad/sec), C: angular distance (AD, in degrees). Note the differences in scale for the AD plots.

There were no significant differences between the mean reaction time (RT) for patients (0.27 s) and for healthy subjects (0.31 s). Healthy subjects managed to execute the circumduction movements at a speed rather close to the target speed of 3.5 rad/s, whereas patients had a lower mean speed of 3.02 rad/s. Healthy subjects showed a delay (AD) of approximately 180 degrees, whereas for patients the delay was approximately 400 degrees (examples of individual polar plots: fig 4A–C). It should be noted, in this respect, that a 400 degree delay implies that circular movement execution lacked more than a full circle i.e., the target cursor is catching up with the subject cursor. Since the results of mean speed and mean AD differed significantly between the two groups of subjects we additionally calculated the coefficient of variation (CV) – which is a better measure for variability than standard deviation when means differ - for both parameters. Speed CV and AD CV instead of speed variability and AD variability were thus used in subsequent comparisons between groups. In patients, there was no correlation between the UPDRS rigidity, or, bradykinesia (upper extremity) scores and any of the kinematic parameters.

#### Radial position (RP)

By comparing the individual polar plots of RP between groups we observed that the RP profile of PD patients was generally circular, whereas the healthy RP profiles were ellipsoidal (example of individual data fig. 4A). This meant that statistical comparison of mean RP between groups was not meaningful because circular and ellipsoidal shapes can have similar mean RP values. The observation of patients having a more circular shaped RP profile was reflected in the correlation values of the RP profile being closer to zero in patients. Additionally, profiles were similar for CW and CCW directions in patients. On the other hand, we did find an effect of direction for RP profile: mean CW RP profile was an ellipse of which the long axis was tilted significantly more to the left than the long axis of the mean CCW RP profile which was tilted to the right (p<0.001). Although mean RP values could not be compared, RP variability could be compared between groups since this is a measure of smoothness of movement execution within an individual subject, independent of the exact shape of the movement. RP variability was significantly larger in patients, indicating that patients had less consistent wrist circumduction trajectories over several trials than healthy subjects (p<0.001, for details see table 2).

**Table 2:** Kinematic data.

Kinematic				Group		Direction	
parameters		HC	PD				
		Mean (SD)	Mean (SD)	*p*	Effect	*p*	Effect
**RP**							
mean		0.26 (0.08)	0.39 (0.10)	-	-	-	-
variability		0.09 (0.03)	0.15 (0.04)	<0.001	PD>HC	-	-
profile	*CW*	−0.04 (0.05)	−0.01 (0.04)	-	-	0.012	CW<CCW
	*CCW*	0.03 (0.05)	0.02 (0.05)				
**Speed**							
mean		3.47 (0.27)	3.02 (0.34)	<0.001	HC>PD	-	-
CV		1.68 (0.70)	2.47 (0.63)	0.003	PD>HC	-	
profile	*CW*	0.05 (0.10)	−0.02 (0.06)	-	-	0.004	CW>CCW
	*CCW*	−0.09 (0.11)	−0.05 (0.10)	-	-		
**AD**							
mean		176.97 (111.85)	439.09 (214.72)	<0.001	PD>HC	-	-
CV		1.12 (0.78)*	0.97 (0.21)	-	-	-	-
profile	*CW*	0.02 (0.02)	0.02 (0.01)*	0.028	PD <HC	0.029	CW>CCW
	*CCW*	0.01 (0.03)	−0.01 (0.01)*			-	-

Statistical results for all kinematic measures. For each parameter mean (SD) is indicated per group and direction. HC: healthy subjects, PD: Parkinson patients. Main effects are indicated by p-values. *  =  median (interquartile range) due to non-normality.

#### Speed

For both mean speed and speed CV a main effect of group was found. Patients were significantly slower than healthy subjects (p<0.001, table 2). Moreover, patients executed the circle movements at a less consistent movement speed (p = 0.003) (fig. 4B). For speed profile, a main effect of direction was observed. The speed profile was tilted more to the right in the CW direction (p = 0.004).

#### Angular distance (AD)

Patients had a larger mean delay (mean AD) than healthy subjects (p<0.001, table 2). For AD profile we found a group effect (p = 0.028, table 2): the healthy subjects showed an AD profile which was tilted more to the right than in patients. Additionally, we found a main effect of direction indicating that AD profile was more tilted to the right in the CW direction (p = 0.029).

### EMG

#### We first generally describe the EMG results

We found that DiffAct was smaller in patients, i.e., lower arm muscles were more constantly active in patients yielding a less specific activity pattern for each muscle along the circle trajectory compared to healthy subjects. This finding quantifies the visually observed differences in the patterns of (filtered, rectified and scaled) EMG in individual healthy subjects and patients (examples in fig. 2). Quantitatively, comparing results for DiffAct and Angle_max_ between and within muscles, patients generally show less modulation of lower arm muscle activity than healthy subjects. Visual inspection of scaled EMG data of individual patients with low values for DiffAct for specific muscles, on the other hand, showed that these patients were still able to use the other (lower arm) muscles in a well-differentiated manner. Additionally, in muscles with the lowest values for DiffAct (which mostly concerned patients), further visual inspection of scaled EMG data showed higher variability in the mean EMG signal compared to the muscles that displayed higher levels of activity modulation (compare examples in fig. 2A vs. 2B). This indicates that the use of the muscle (in terms of the amount of EMG activity) in a particular phase of wrist circumduction varies more from circle to circle in patients than in healthy subjects; muscles may not be used in the same manner for each circle movement that is performed. There were no correlations between DiffAct and the UPDRS rigidity, or, bradykinesia (upper extremity) scores of the patients.

#### Differentiated muscle activity (DiffAct)

**Figure 5:**
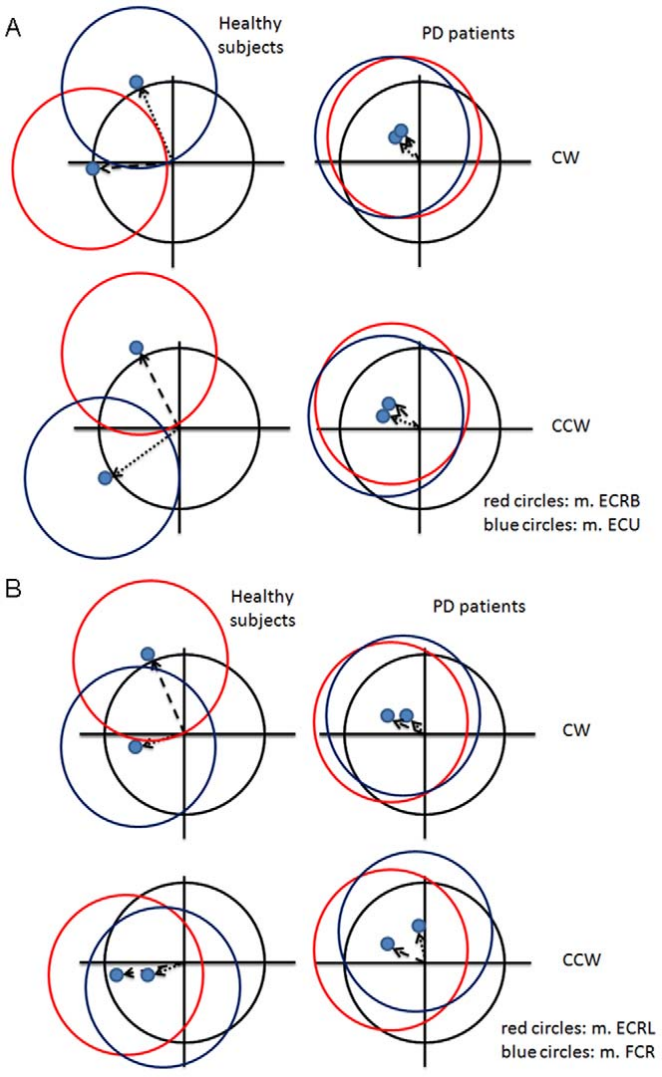
Schematic overview of mean EMG activity along the circumduction trajectory. Differentiated muscle activity was described using the center of the fitted EMG ellipses (blue dots). Mean results are displayed per group (separately for muscle and direction). The black circle represents fictive EMG activity in a situation of consistent activity along the trajectory. A–B: the two coloured circles (blue and red) represent the EMG ellipses (ellipses simplified to circles) of two different muscles showing the activity pattern along the circumduction trajectory. A: blue = m. Extensor carpi ulnaris (m. ECU); red = m. Extensor carpi radialis brevis (m. ECRB), B: blue = m. Flexor carpi radialis (m. FCR); red = m. ECRL.

DiffAct was smaller in patients compared to healthy subjects (p = 0.001, see table 3). Additionally, we found a main effect of muscle (p<0.001). Post-hoc comparisons between muscles showed that m. ECRB was used in a more differentiated fashion (higher DiffAct) than m. ECRL (p = 0.022), m. FCR (p = 0.001) and m. FCU (p = 0.003). Furthermore, we found that m. ECU was used significantly more differentiatedly than m. ECRL (p = 0.017), m. FCR (p = 0.002) and m. FCU (p<0.001) (details are shown in table 3, for mean EMG activity differentiation patterns in all muscles and both groups see schematic overviews in fig. 5).

**Table 3:** EMG data: DiffAct.

EMG	Group		Muscle	
Differentiated muscle use	HC	PD	Post-hoc	
(CW + CCW)	mean (SD)	mean (SD)	*p*	Effect
**m. ECRL**	0.04 (0.02)	0.01 (0.02)*		
**m. ECRB**	0.06 (0.03)	0.02 (0.03)*	0.022	ECRB>ECRL
			0.001	ECRB>FCR
			0.003	ECRB>FCU
**m. FCR**	0.03 (0.01)	0.02 (0.01)		
**m. ECU**	0.06 (0.02)	0.02 (0.04)*	0.017	ECU>ECRL
			0.002	ECU>FCR
			<0.001	ECU>FCU
**m. FCU**	0.03 (0.02)	0.02 (0.01)		

Results for the EMG parameter differentiated muscle activity (DiffAct). For each muscle the mean (SD) is shown per group. HC: healthy subjects, PD: Parkinson patients. The results of the post-hoc tests for main effect of muscle are indicated by p-values. *  =  median (interquartile range) due to non-normality.

#### Angle of maximal muscle activity (Angle_max_)

We found that healthy subjects had a larger Anglemax than patients (p = 0.003). In addition, we found an interaction effect between direction and muscle (p<0.001) and an interaction effect between direction and group (p = 0.016). Further analyses of the interaction effects indicated that in healthy subjects, Anglemax for the muscles on the radial side of the arm was larger in CW than in CCW direction while the opposite was found for the CCW direction. For both flexors differences between Anglemax in CW and CCW direction, on the other hand, were less clear. Moreover, for patients the within-muscle differences between directions were less evident (details shown in table 4; for overview of muscle activity patterns see fig. 5A–B). This is likely due to the fact that differentiated muscle activity (DiffAct) is much smaller and less defined in patients, implying that the angle at which muscle activity is maximum (Anglemax) is more variable between patients.

**Table 4:** EMG data: Angle_max._

EMG	Direction CW		Direction CCW	
Angle_max_	HC	PD	HC	PD
	Mean (SD)	Mean (SD)	Mean (SD)	Mean (SD)
**m. ECRL**	3.36 (0.44)	2.79 (1.00)	2.51(0.96)	2.24 (1.25)
**m. ECRB**	3.31 (0.49)*	2.10 (1.04)	2.01 (1.07)	2.69 (1.03)
**m. FCR**	3.10 (1.07)	2.16 (1.44)	2.54 (1.38)	1.66 (1.10)
**m. ECU**	1.83 (0.65)*	1.80 (1.29)	4.02 (1.07)*	2.92 (1.20)
**m. FCU**	2.55 (1.14)*	2.30 (1.22)	2.81 (1.27)	1.97 (0.81)

Results for the EMG parameter Angle_max_. For each muscle the mean (SD) is displayed separately for each group and direction. HC: healthy subjects, PD: Parkinson patients. *  =  median (interquartile range) due to non-normality.

#### Differentiated muscle use related to kinematic parameters

**Figure 6:**
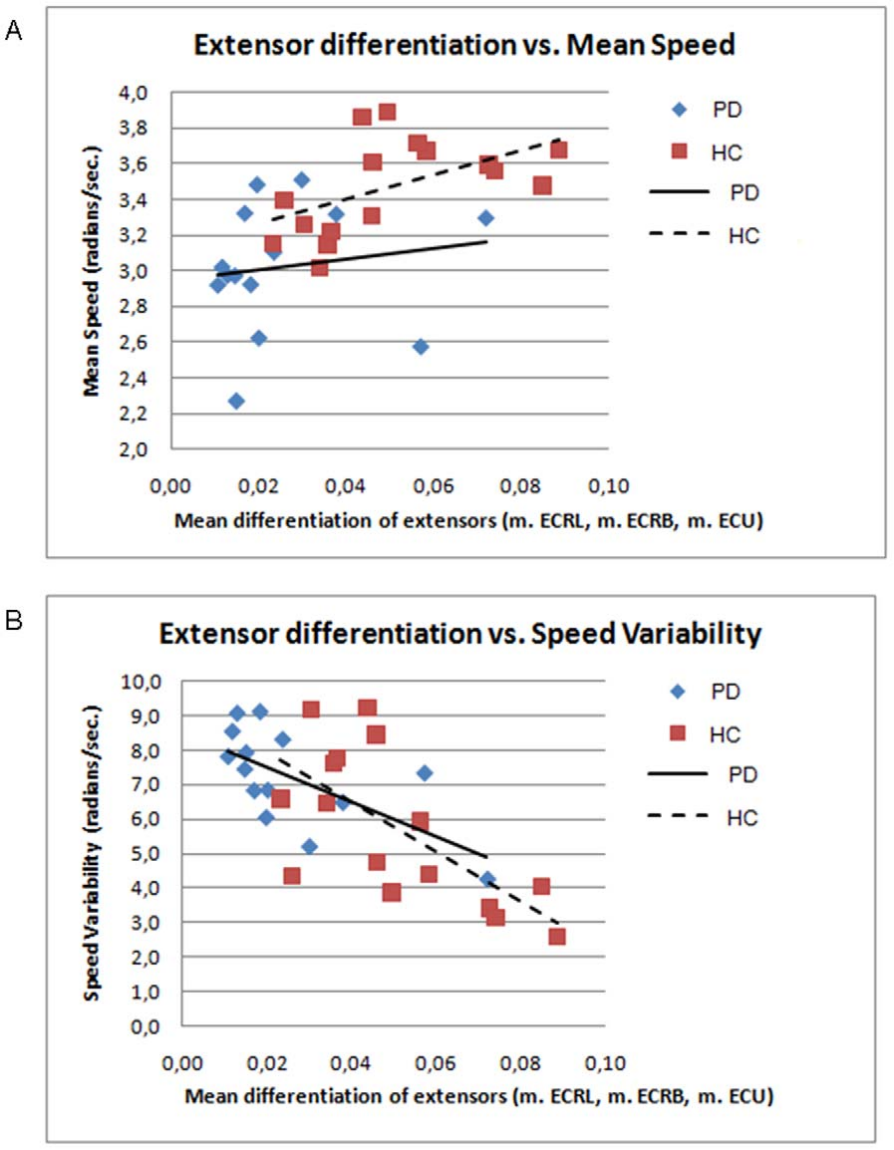
Correlations between the mean differentiated muscle activity (DiffAct) of the three extensor muscles and speed parameters. A: DiffAct of extensors vs. mean speed, blue diamonds: PD patients, red squares: healthy subjects; B: Differentiated muscle use of extensors vs. speed variability, blue diamonds: PD patients, red squares: healthy subjects. Linear regression lines are indicated for both groups.

The relation between the extent of differentiated muscle use and the quality of movement execution was investigated by calculating correlations between DiffAct and kinematic parameters (fig. 6A–B). Since the extensor muscles had the largest DiffAct, we used the mean DiffAct of the three extensor muscles to test for correlations with mean speed and speed variability (separately within each group). Here, variability instead of CV is used as these correlations were performed within groups meaning that a correction for differences in mean speed was not required. The result indicates that in healthy subjects more differentiated extensor muscle use was associated with higher mean speed (r2 = 0.449, p = 0.004). This correlation was not found for patients. Additionally, we found that, for both healthy subjects and patients, more extensor differentiation resulted in lower speed variability (r2 = 0.280, p = 0.035 and r2 = 0.391, p = 0.017, respectively).

## Discussion

The present study investigated the relation between activity patterns of gradually adjusting muscles and the quality of continuous movement performed by the wrist. As PD is a disease primarily affecting *cerebral* motor control, the observed changes of these movement parameters in patients, compared to healthy subjects, provide a first step to gain insight into the mechanism of how changes in cerebrally coded modulation of movement initiation and inhibition influence the execution of continuous movement by the musculoskeletal system in these patients. Using a continuous wrist circumduction task, EMG and kinematic parameters showed that PD patients had less differentiated muscle activity and impoverished task performance - as reflected in lower movement speed and higher movement variability - compared to healthy subjects. Since the mean age of the healthy group of subjects was higher than the mean age of the patient group, the possibility that our findings are related to aging effects can be virtually excluded [30], [31].

Differentiated muscle use was quantified using EMG. We found that PD patients had a less differentiated muscle activity pattern of lower arm muscles when compared to healthy subjects. Moreover, healthy subjects used extensor muscles more differentiatedly than flexor muscles, while this was less evident in patients. The observation that extensor muscles showed clearer modulation of activity during the task was further underlined by the finding that only for extensors the Angle_max_ was different for the two directions of circle movement. These findings suggest a specific role for extensors in wrist circumduction tasks. Within the patient group, there were fewer differences in Angle_max_ between extensors and flexors. From these results we inferred that patients are less capable of modulating muscle activity of particularly the appropriate wrist extensor muscle groups. This conclusion is consistent with a study of Robichaud et al. who found that patients are more impaired for the extensors than for the flexors during a rapid flexion-extension task [32].

Our finding of a less differentiated muscle activity pattern during a continuous movement task indicates impaired adjustment of muscle activity in patients. However, despite reduced differentiation of muscle activity, patients were still able to execute wrist circumduction, probably due to generating small differences in activity between muscles. More detailed visual inspection of individual mean scaled EMG indeed showed that, even in patients with the lowest values for differentiated muscle activity, muscles maintained some extent of differentiation. This illustrates that differentiated muscle activity is, even on a small scale, essential for rotating the wrist joint. Indeed, without any muscle differentiation there would only be constant muscle contraction and no movement. One may speculate that patients with advanced PD will not be able to execute the circle movement task due to further loss of differentiation. As a result, muscles will be equally active along the circle trajectory and smooth wrist rotation will no longer be possible.

The reduced differentiated muscle activity that we found in patients probably contributes to the decline of movement performance in this group as reflected in lower speeds (resulting in a greater delay) and greater movement variability. These results could not be explained by a delay in movement onset since there was no significant difference in RT between patients and healthy subjects. Lower speed in patients is in line with the phenomenon of bradykinesia [10] and suggests that a decreased ability to phase synergistic muscle activity has a negative effect on speed. Indeed, we found a correlation between differentiated muscle use and movement speed, although only in healthy subjects. On the other hand, there was a negative relation between speed variability, which is commonly observed to be increased in PD [33]–[38], and differentiated muscle use in both healthy subjects and patients. This indicates that in order to perform purposeful movement, smooth adjustments of distinctive muscles is a prerequisite. In healthy subjects, we were able to quantitatively describe differentiated use of lower arm muscles during a movement task with specific timing and spatial constraints, reflecting the most efficient way to accomplish purposeful movement performance. This is consistent with the previous work of Hoffman and Strick who found that specific combinations of muscles were used for different directions in step-tracking tasks [28]. Hence, a specialized, fine-tuned muscle activity pattern seems to be necessary to optimize (smooth) movement performance.

The observed reduction in differentiation of activity of opposite muscles in PD may reflect the consequence of altered neuronal activity implicated in initiation and inhibition on cerebral level for movement output. It has been found that modulation of initiation and inhibition is, at least partially, facilitated by the BG that selectively inhibit undesired motor activity [5], [39]. Additionally, the cerebellum may be involved; synergistic muscle tuning requires feedforward processing to adjust planned movements of several muscle groups [40], [41] and several imaging studies have found task-related changes in cerebellar activation in PD [42], [43]. Considering the disturbed functionality of the BG and interconnected circuitry in PD [7], [9]–[12], [44], impaired gradual adjustment of muscle activity might be related to a declined ability to select appropriate muscles [5], [39]. On the other hand, the impaired gradual adjustment of co-active muscles might also be related to a decrease in scaling of the magnitude of co-active muscles [14] or a diminished ability to time selection of appropriate muscle activity. To further elucidate the relation between impaired cerebrally coded task performance and final execution by the musculoskeletal system, a successful strategy could be the application of continuous movement tasks analyzed with concurrent electrophysiological and functional imaging techniques.

We conclude that during wrist circumduction, PD patients have a reduced ability to gradually adjust muscles. This is reflected in reduced differentiated muscle activity, resulting in a decline of overall movement performance. The reduction of differentiated muscle activity of opposite muscles illustrates a failure of selective control mechanisms that normally optimize muscle activity during continuous movement. Given the eminent place of striatal dysfunction in PD, we hypothesize that impaired modulation of initiation and inhibition of movement might be caused – at least to some extent - by impaired selection of appropriate muscles at the level of the basal ganglia.
